# Electrochemical and thermal detection of allergenic substance lysozyme with molecularly imprinted nanoparticles

**DOI:** 10.1007/s00216-023-04638-2

**Published:** 2023-03-11

**Authors:** Pankaj Singla, Sarbjeet Kaur, Oliver Jamieson, Amy Dann, Saweta Garg, Clare Mahon, Robert D. Crapnell, Craig E. Banks, Inderpreet Kaur, Marloes Peeters

**Affiliations:** 1 School of Engineering, Newcastle University, Merz Court, Claremont Road, Newcastle Upon Tyne, NE1 7RU UK; 2 Department of Chemistry, Centre for Advanced Studies, Guru Nanak Dev University, Amritsar, Punjab 143005 India; 3 Faculty of Science and Engineering, Manchester Metropolitan University, John Dalton Building, Chester Street, Manchester, M1 5GD UK; 4 Department of Chemistry, Durham University, Lower Mount Joy, South Road, Durham, DH1 3LE UK

**Keywords:** Molecularly imprinted polymer nanoparticles (nanoMIPs), Biomimetics, Protein sensing, Electrochemistry, Heat transfer method, Lysozyme

## Abstract

Lysozyme (LYZ) is a small cationic protein which is widely used for medical treatment and in the food industry to act as an anti-bacterial agent; however, it can trigger allergic reactions. In this study, high-affinity molecularly imprinted nanoparticles (nanoMIPs) were synthesized for LYZ using a solid-phase approach. The produced nanoMIPs were electrografted to screen-printed electrodes (SPEs), disposable electrodes with high commercial potential, to enable electrochemical and thermal sensing. Electrochemical impedance spectroscopy (EIS) facilitated fast measurement (5–10 min) and is able to determine trace levels of LYZ (pM) and can discriminate between LYZ and structurally similar proteins (bovine serum albumin, troponin-I). In tandem, thermal analysis was conducted with the heat transfer method (HTM), which is based on monitoring the heat transfer resistance at the solid–liquid interface of the functionalized SPE. HTM as detection technique guaranteed trace-level (fM) detection of LYZ but needed longer analysis time compared to EIS measurement (30 min vs 5–10 min). Considering the versatility of the nanoMIPs which can be adapted to virtually any target of interest, these low-cost point-of-care sensors hold great potential to improve food safety.

**Graphical Abstract:**

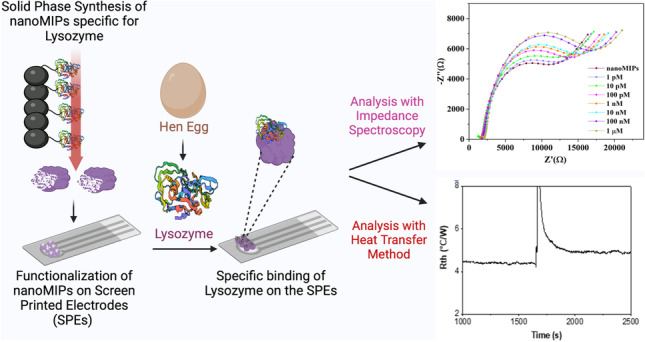

**Supplementary Information:**

The online version contains supplementary material available at 10.1007/s00216-023-04638-2.

## Introduction

Lysozyme (LYZ), also known as muramidase or N-acetylmuramic hydrolase, is a 14-kDa cationic protein. It damages or kills bacteria via lysing peptidoglycan in the bacterial cell wall and disrupting their cell membrane [1]. Due to its bactericidal properties, lytic activation, and low molecular weight, LYZ has been widely applied for medical treatment and in the food industry to act as an anti-bacterial agent [2]. Levels in bodily fluids range from µg L^−1^ levels in urine to 2.9 mg L^−1^ in serum and 1.65 g L^−1^ in tears, with increased serum levels being non-specific indicators of leukaemia and liver and kidney diseases [3, 4]. However, LYZ can trigger allergic reactions even when present in trace amounts and 32% of individuals with egg allergy are sensitized to this compound [5]. For instance, the recommended maximum level of LYZ to add during the production process of wine is 0.5 g L^−1^ [6]. Thus, the accurate determination of LYZ in clinical and food samples in a fast and low-cost manner is of paramount importance from a medical and food safety perspective. Moreover, LYZ has long been used as a prominent model system for systematic studies on protein–protein interactions and to evaluate new detection methods.

Traditional detection of LYZ via enzyme-linked immunosorbent assays (ELISA) or chromatographic methods are either expensive or time-consuming, and cannot be used for fast in situ quantification [7, 8]. Therefore, there has been a drive towards portable devices that can facilitate fast and low-cost measurements. In particular, optical and electrochemical biosensors combined with specific recognition elements have attracted increasing attention [9]. Coupling of surface-enhanced Raman spectroscopy with biomimetic receptors and chemometric analysis have demonstrated high sensitivity and selectivity for the detection of LYZ [10]. However, these methods cannot be miniaturized and therefore do not offer on-site detection. Electrodes modified with nanomaterials or polymeric films for sensing of proteins offer fast analysis times and high sensitivity and can be easily integrated into portable devices. An electrode modified with a composite of acrylic acid and hollow TiO_2_ spheres and embedded aptamers as recognition elements was constructed [11]. Electrochemical impedance spectroscopy (EIS) results demonstrated that the aptasensor was able to measure LYZ with a limit of detection of 1.04 pM and a dynamic range between 0.05 and 100 µg L^−1^. Aptasensors have been shown to be a promising technology for food allergen detection with an all-field search in Scopus using “allergens” and “aptamers” showing an increasing trend with 789 results in the 2016–2020 period [12]. Despite this increasing interest, aptamers can suffer from limited stability and specificity in complex matrices, which remains an issue for industrial development [13].

Molecularly imprinted polymers (MIPs), porous materials containing high-affinity binding sites for the analyte of interest, can rival natural recognition elements in terms of affinity but possess superior thermal and chemical stability [14, 15]. There are several reports of MIPs for LYZ in the literature including MIP gels using acrylamide and methacrylic acid as functional monomers which facilitated complete removal of lysozyme from egg white [16]. However, MIPs that are traditionally prepared via bulk polymerization exhibit problems with diffusion limitations in the 3D matrix and resulting slow kinetics due to the large size of proteins [17]. To overcome these limitations, nanoscale and surface imprinting strategies have been employed which require less protein template to reduce synthesis cost and improve accessibility of the recognition sites [18, 19]. Surface protein imprinted core–shell particles combined with reversible additional-fragmentation chain transfer polymerization enables to improve the kinetics of binding and enhance control over the surface binding sites [20]. As determined by high-performance liquid chromatography (HPLC) with UV–vis detection, these imprinted particles could bind 5.6 mg protein/mg material and could selectively recognize LYZ from a four-protein mixture and an egg white sample. This concept can be further explored when depositing thin MIP layers onto iron oxide particles to facilitate easy separation of LYZ from a complex solution by applying an external magnetic field [21]. The complication of using molecularly imprinted nanoparticles in the diagnostics and therapeutic sector is their biocompatibility and their integration into portable devices [22]. In this respect, the preparation of molecularly imprinted nanoparticles (nanoMIPs) holds great potential. Water-soluble nanoparticles (NPs) were formed by self-assembling γ-glutamic acid with 3-aminothiophene followed by electropolymerization to generate a conductive polymer network. A MIP-based biosensor was produced which could detect LYZ in the 1 × 10^−10^ to 1 × 10^−5^ g L^−1^ range using differential pulse voltammetry as read-out strategy [23]. These nanoparticles can be multifunctional and a “catch and release” system was developed where nanoMIPs can release LYZ, which was chosen as a model protein, upon changing the temperature [24].

Besides biocompatibility, it is essential to have a scalable and straightforward nanoMIP production process. Solid-phase imprinting is an innovative technique for the medium-scale fabrication of nanoMIPs with a homogenous distribution of binding sites and affinity similar to monoclonal antibodies due to the use of the solid phase as affinity medium [25, 26]. These nanoMIPs have been used to construct highly selective and specific optical, thermal, and electrochemical sensors for proteins [27–29]. In this manuscript, we use solid-phase imprinting to develop nanoMIP-based sensors for the electrochemical and thermal detection of LYZ. These nanoMIPs will be electrografted onto screen-printed electrodes (SPEs), which have high potential for commercial use since they are disposable and low cost, and exhibit high batch-to-batch inconsistency [30]. We demonstrate that it is possible to detect LYZ with high accuracy and selectivity at relevant concentrations using either electrochemical detection or with an in-house developed thermal device. Since the nanoMIPs are highly versatile and adapting this for other targets is straightforward, the technology presented will serve as a platform for food allergen detection.

## Experimental section

### Reagents

Glass beads (53–106-μm diameter, Spheriglass 2429 CP00) were purchased from Blagden Chemicals. N-Isopropylacrylamide (NIPAM), N,N′-methylenebisacrylamide (BIS), N-tert-butylacrylamide (TBAm), N-(3-aminopropyl)methacrylamide hydrochloride (APMA), acrylic acid (AAc), and N,N,N′,N′-tetramethylethylenediamine (TEMED), (3-aminopropyl)trimethoxysilane (APTMS), glutaraldehyde (GA), LYZ (from chicken egg white), dialysis cartridges (Vivaspin® 20, 3 k-Da MWCO polyethersulfone) and Supelco polypropylene solid-phase extraction tubes (60 mL), potassium chloride (KCl), potassium hexacyanoferrate(II) trihydrate, and potassium ferricyanide(III) were purchased from Sigma-Aldrich (Gillingham, UK). PierceTM BCA Protein Assay Kit, ammonium persulfate (APS), methanol, acetone, acetonitrile, sodium hydroxide (NaOH), N-hydroxysuccinimide (NHS), 4-aminobenzoic acid, hydrochloric acid (33%, HCl), 1-ethyl-3-(3-dimethylaminopropyl) carbodiimide (EDC), and sodium nitrite were purchased from Fisher Scientific UK Ltd (Loughborough, UK) and used without purification. All chemicals and solvents were high-performance liquid chromatography (HPLC)/analytical grade and were used without any further purification. PBS solutions were prepared with deionized (DI) water with resistivity of ≥ 18.2 MΩ cm).

### NanoMIP synthesis

#### Preparation of lysozyme-derivatized glass beads

Sixty grammes of glass beads was incubated in 2 M NaOH (24 mL) for 20 min for their activation and then washed with double-distilled water (10 times with 100 mL) until the pH of the washed solution was around 7.4. Subsequently, glass beads were rinsed twice with acetone (100 mL) and dried at 80 °C for 2 h and then immersed in a 24 mL solution of 2%, vol/vol APTMS in anhydrous toluene for 12 h for silanization. Afterwards, the beads were washed with 100 mL (eight times) of acetonitrile and followed by 100 mL (two times) of methanol. Successively, silanized glass beads were incubated for 2 h in 7% (vol/vol) glutaraldehyde (GA) solution in 10 mM phosphate-buffered saline (PBS) solution of pH = 7.4 (0.4 mL of solution per gram of beads). Subsequently, GA-modified glass beads were washed with deionized water (eight volumes) in a Buchner funnel under vacuum. Twelve milligrammes of LYZ (0.5 mg mL^−1^) was added to the GA-modified beads in 24 mL of 10 mM PBS (pH = 7.4) solution and was incubated overnight. The LYZ cross-linked glass beads were washed with doubled-distilled water (5 × 100 mL) and dried under vacuum. Bicinchoninic assay was performed to confirm the cross-linking of LYZ on GA-modified beads and used straightway for the nanoMIPs synthesis.

#### Synthesis of nanoMIPs

A protocol documented in literature [25] was followed to manufacture nanoMIPs with high specificity and selectivity for LYZ. Briefly, 39 mg of NIPAM, 2 mg of BIS, 33 mg of TBAm dissolved in 1 mL of ethanol, 5.8 mg of APMA, and 2.2 μL of AAc were dissolved in 100 mL of PBS (pH = 7.4). The total monomer concentration was 6.5 mM at this stage. The monomer mixture solution was sonicated under vacuum for 10 min, and then purged with N_2_ for 30 min. Eventually, 60 g of LYZ cross-linked glass beads was introduced to the solution, and polymerization was initiated by adding mixture containing 800μL of APS aqueous solution (60 mg/mL) and 24 μL of TEMED. The reaction mixture vessel was further flushed with N_2_ through hole of port cap, and subsequently sealed. The mixture was kept for 4 h at room temperature (RT) for polymerization after which it was poured into a solid-phase extraction tube (60 mL) consisting of a frit (20 μm porosity). The removal of low affinity nanoMIPs, polymer, and unreacted monomers was achieved by washing with distilled water (9 × 20 mL) at RT. Subsequently, 20 mL of DI water pre-warmed at 65 °C was poured into the SPE and was placed in a water bath at 65 °C for 15 min. This step was repeated five times until ~ 100 mL of high-affinity nanoMIP solution was collected. Concentrated nanoMIPs were obtained by drying them in oven for 24 h at 60 °C to evaporate water, after which they were rehydrated with a given amount of water according to the concentration needed. Five washes (10 mL) with deionized water were performed using the dialysis cartridge (Vivaspin® 20, 3-kDa MWCO polyethersulfone) and the obtained nanoMIPs were re-suspended in 50 mL of deionized water. The size distribution of resulting nanoMIPs was characterized by dynamic light scattering (DLS) experiments and transmission electron microscopy (TEM). DLS experiments were performed with a Malvern Zetasizer Nano ZS to measure the hydrodynamic diameter (*D*_*h*_) at a temperature of 25 ± 0.1 °C. The instrument used a scattering angle of 173° and had a laser wavelength of 632.8 nm.

For SEM measurements, firstly, samples were drop-casted on the glass slides (1 × 1 cm) and left for 12 h for evaporation of water. Images were recorded using a Tescan Vega 3LMU scanning electron microscope from Tescan Orsay Holding (USA). Samples were mounted onto aluminium SEM pin stubs (12-mm diameter).

### Electrode functionalization and electrochemical measurements

The graphite screen-printed electrodes (SPEs) with a diameter = 3.1 mm were fabricated in-house with appropriate stencil designs using a microDEK 1760RS screen-printing machine (DEK, Weymouth, UK). Firstly, a carbon-graphite ink formulation (Product Code: C2000802P2; Gwent Electronic Materials Ltd, UK) was printed onto a polyester (Autostat, 250 micron thickness) substrate. This layer was then cured in a fan oven at 60 °C for 30 min. Finally, a dielectric paste (Product Code: D2070423D5; Gwent Electronic Materials Ltd, UK) was then printed onto the polyester substrate to cover the connections. After curing 60 °C for 30 min, the screen-printed electrodes are ready to be used. The reproducibility of the batch of screen-printed electrode was found to correspond to less than 4.2% RSD towards the redox probe, [Ru(NH_3_)]^2+/3+^/0.1 M KCl. This was followed by curing at 60 °C for 30 min with a dielectric material, which was used to define the rectangular shape of the SPE for easy handling [31]. These SPEs had an average connection length of 32 mm with a working electrode resistance of 2.16 ± 0.06 kΩ [31]. The LYZ-nanoMIPs were functionalized onto SPEs using an electrografting procedure established in our lab [32]. EIS and cyclic voltammetry (CV) were performed with a Reference 3000™ potentiostat/galvanostat (Gamry Instruments, USA). All electrochemical procedures were carried out with a three-electrode system comprising a SPE as the working electrode, a platinum wire as auxiliary electrode, and Ag/AgCl electrode (sat. KCl) serving as a reference electrode.

All the measurements were performed in 1 mM [Fe(CN)_6_]^3−/4−^ (1:1) mixture in 0.1 M KCl in PBS. CV studies were performed in the potential range from − 0.2 to 0.6 V with a scan rate of 50 mV/s. EIS measurements were performed in a wide frequency range from 0.02 Hz to 100 kHz with an amplitude of 10 mV at open circuit potential. To construct dose–response curve, a SPE functionalized with nanoMIPs was exposed to PBS solutions with increasing concentrations of LYZ (0–1 mM). Measurements to construct dose–response curves to monitor the corresponding charge transfer resistance (R_CT_) values at certain concentrations of LYZ were calculated by fitting equivalent circuits to Nyquist plots corresponding to each EIS measurement.

### Thermal measurements

**Scheme 1:**
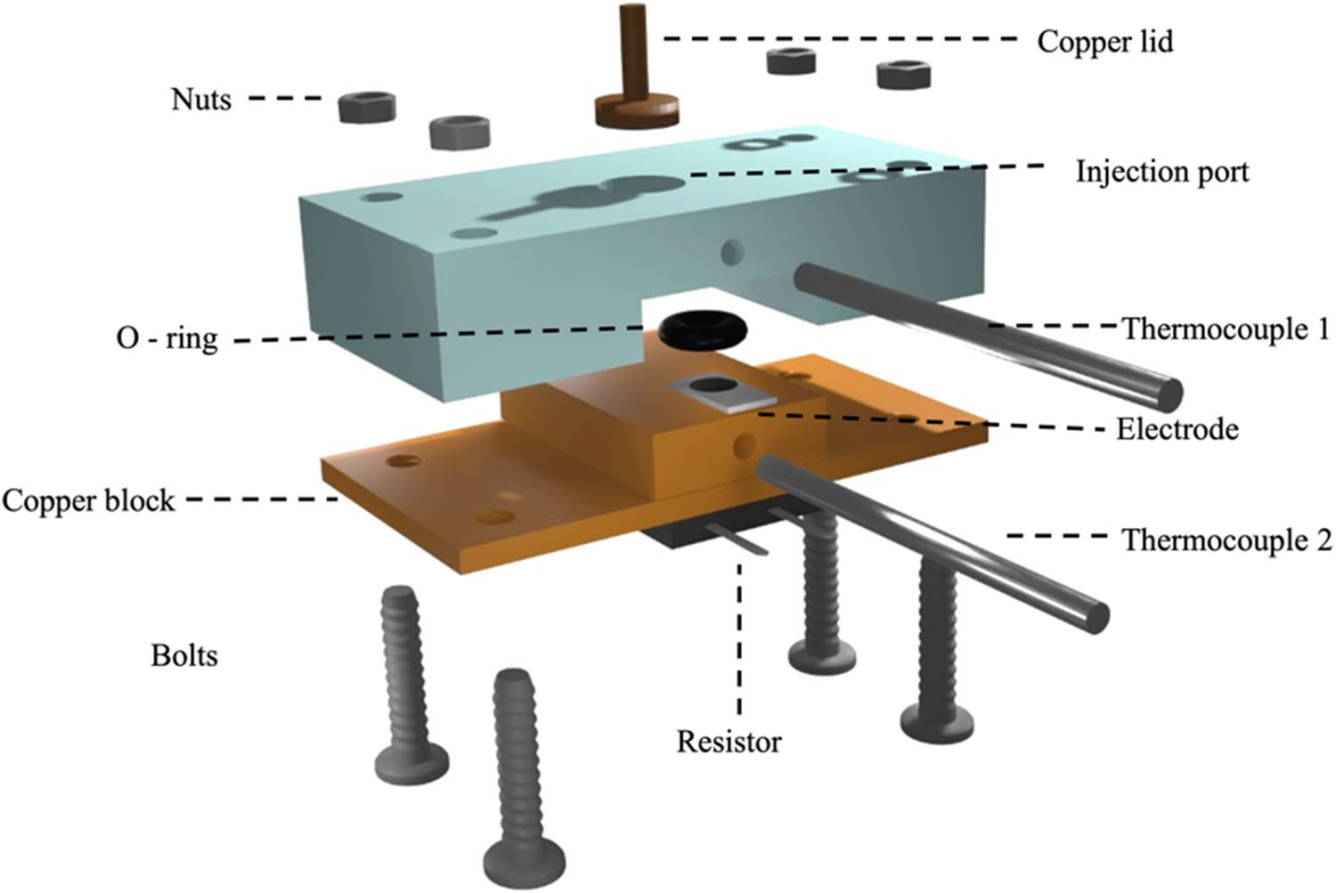
Schematic design of the fully assembled measurement chamber, which includes the 3D-printed cell, a copper lid, the functionalized SPE, and a copper heat sink to which a resistor is attached to control the temperature. The thermocouples measure the temperature of the copper and the temperature of the liquid inside the cell

A measurement chamber with an internal volume of 150 µL, as designed in [33], was 3D-printed using an Anycubic Photon printer (Shenzhen, China). Functionalized SPEs were mounted onto a copper heat sink, which was sealed off with an O-ring and a copper lid to prevent leakage, and subsequently placed on top of the measurement chamber (Scheme 1).

The measurement set up was coupled to an in-house designed thermal device [34]. For all experiments, the thermal measurement device was controlled using LabView software and a proportional-integral-derivative (PID) controller attached to a power resistor (22 Ω). The PID parameters were optimized for this experiment and included P = 1, I = 40, and D = 0.3. The temperature of the copper heat sink was kept at 37 ± 0.02 °C via the PID controller. The temperature of the liquid in the measurement cell (T_2_) was recorded via a type-K thermocouple (RS Components, City, UK) placed at 1.7 mm above the electrode surface. The thermal resistance (R_th_, °C/W) can then be calculated via dividing the temperature gradient (T_2_ − T_1_) by the power input (P) required to keep the heat sink at the desired temperature.

1 $${{\varvec{R}}}_{{\varvec{t}}{\varvec{h}}}=\frac{({{\varvec{T}}}_{1}-{{\varvec{T}}}_{2})}{{\varvec{P}}}$$

First, a PBS solution (150 µL) was introduced into measurement cell containing a MIP-functionalized SPE and allowed to stabilize until a stable temperature signal was recorded. Subsequently, this PBS solution was withdrawn, and a PBS solution (150 µL) spiked with a known concentration of LYZ was added. The impact on the R_th_ was determined when the signal had stabilized (after 15–20 min), with binding of the lysozyme to the MIP layer expecting to increase the thermal resistance as determined by the pore blocking model [35]. LYZ concentrations were measured in a wide range starting at 10 pM, to 1 nM, 0.1 µM, 10 µM, and finally 1 mM. Each MIP-functionalized SPE was used for two measurements. The limit of detection (LoD) was determined via the three-sigma method.

### Proof-of-application impedimetric detection in an egg white sample

The proposed impedimetric sensor was applied to detect LYZ in an egg white sample. The following steps were taken to prepare the hen egg sample for use: first, the egg white and yolk were separated, and then, the egg white was diluted with 10 mM PBS buffer solution (pH 7.5) in a 1:50 ratio. The sample was further diluted to 5000 times and EIS was employed to determine the concentration of LYZ. The hen eggs were obtained from the local Coop supermarket (https://www.coop.co.uk/our-suppliers/farmers/eggs).

## Results and discussion

### NanoMIP and functionalized electrode characterization

**Scheme 2:**
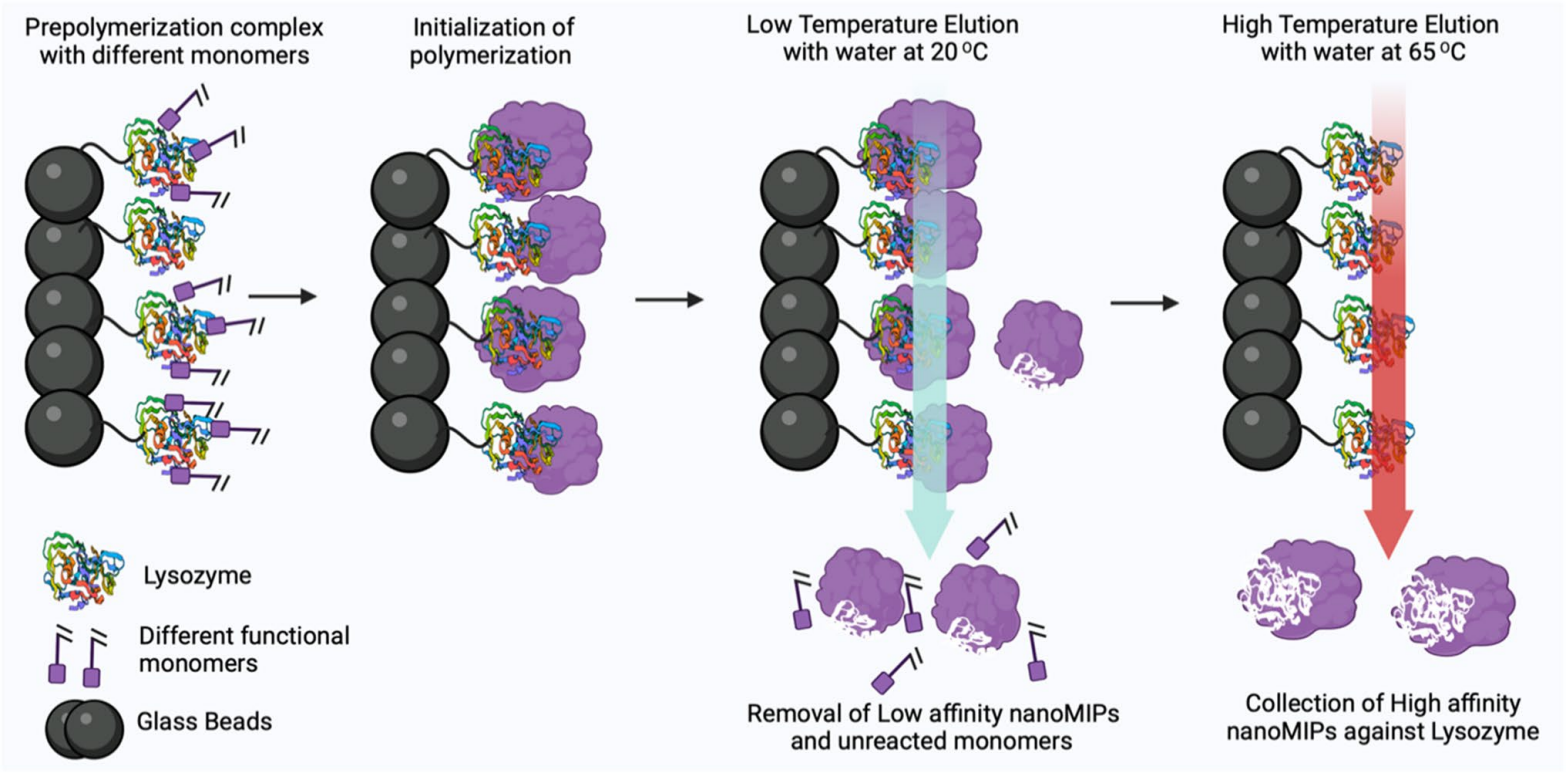
Solid-phase imprinting approach to produce high-affinity nanoMIPs for lysozyme

As LYZ is a small protein with low cost and a monomer structure, we decided to imprint with the whole protein rather than selecting an epitope. The monomer mixture used to manufacture the nanoMIPs was based on previous literature reports and covered a wide range of non-covalent interactions to promote binding, with negatively charged AAc and positively charged APMA able to interact with charged LYZ residues. Furthermore, NIPAM can facilitate hydrogen bonding with lysozyme and hydrophobic TBAm can interact with corresponding hydrophobic amino acids [1]. The synthesis process for nanoMIPs for LYZ is shown in Scheme 2.

**Fig. 1:**
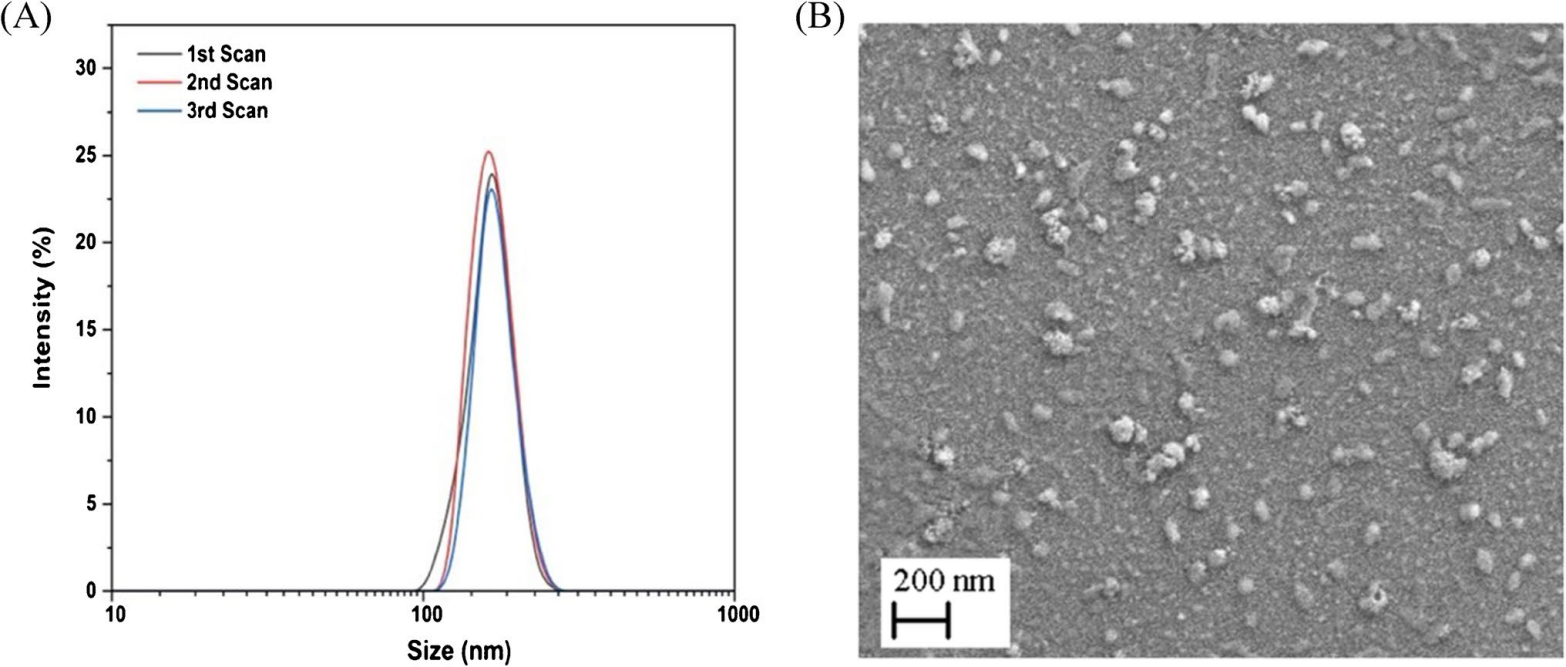
(**A**) Particle size distribution (intensity % vs size nm) for the LYZ specific nanoMIPs as determined by DLS, average hydrodynamic diameter (D_h_) 158 nm (*n* = 3). (**B**) SEM images of the nanoMIPs on the surface, demonstrating particles are 40–s100 nm when measured in the dry state

Following complexation of LYZ with the aforementioned monomer mixture, polymerization was carried out via initiation by APS and TEMED. Low-affinity nanoMIPs and unreacted monomers were removed by filtration using a SPE tube at low temperature, whereas at high temperature (65 °C) nanoMIPs with high affinity were collected. The obtained nanoMIPs were dried at 60 °C to determine their concentration, which was 160 µg mL^−1^. DLS shows that the nanoMIPs were monodisperse (PDI = 0.24) and possessed a hydrodynamic diameter of ~ 158 nm (Fig. 1A). SEM images confirmed that spherical nanoMIPs were formed with an average size range from 40 to 100 nm. It was expected that the size found by DLS was larger as bigger particles have a bigger contribution in the measured intensity. Moreover, monomers used in the synthesis including AAc and NIPAM exhibit significant swelling in liquid and therefore have a bigger size in DLS (conducted in liquid) compared to SEM (conducted in dry state).

### Electrochemical experiments and detection of LYZ

**Fig. 2:**
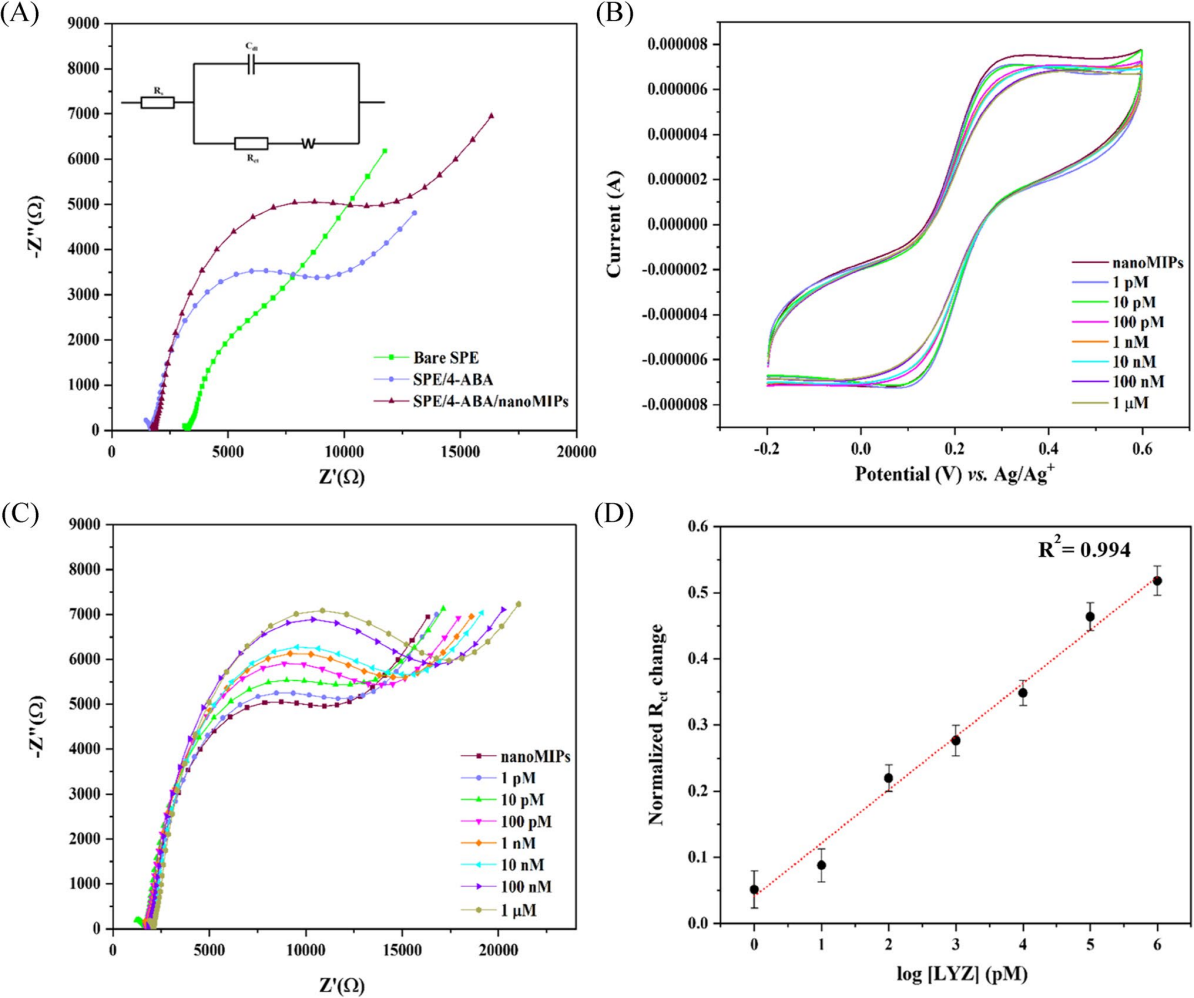
(**A**) EIS measurements with the Nyquist plots for the bare SPE, 4-ABA electrografted onto the SPEs, and nanoMIPs covalently immobilized onto the SPE. (**B**) Representative cyclic voltammograms of nanoMIP-immobilized SPE in the presence of various concentrations of LYZ (1 pM to 1 µM) in PBS with 1 mM ferrocyanide, 1 mM ferrocyanide, and 0.1 M KCl. (**C**) Nyquist plots for nanoMIP-functionalized SPE in the presence of various concentrations of LYZ (1 pm to 1 µM) in PBS with 1 mM ferrocyanide, 1 mM ferrocyanide, and 0.1 M KCl. (**D**) Calibration curve of R_ct_ vs different LYZ concentrations. Each experiment was performed in triplicate

LYZ-specific nanoMIPs (c = 300 µg mL^−1^) were chemically cross-linked onto the surface of SPEs using a previously reported electrografting approach. In short, this involves electrografting of 4-aminobenzoic acid (4-ABA) followed by an EDC/NHS-mediated coupling of the carboxylic groups of 4-ABA to the amine groups present in the nanoMIPs. Following the electrografting, the SPEs were washed with double-distilled water and used for electrochemistry measurements after drying. EIS was utilized as a quick and straightforward technique to follow the surface functionalization and nanoMIP attachment using charge transfer resistance (R_CT_) values of the bare and functionalized SPE (Fig. 2A). A simple Randles circuit (Fig. 2A) was fitted to the respective Nyquist plots to determine the change in transfer resistance (R_CT_) throughout the functionalization process. In Fig. 2B, cyclic voltammetry was performed on the functionalized SPEs. Measurements were done with PBS buffered solutions and subsequently with PBS buffered solutions spiked with increasing concentrations (1 pM to 1 μM) of LYZ. The same experiment was performed where EIS was used to monitor the influence of LYZ concentration on the R_CT_ value of the nanoMIP-functionalized SPE (Fig. 2C). Finally, a dose–response curve is given in Fig. 2D, which demonstrates the dependence of the R_CT_ value depending on the LYZ concentration.

Bare SPEs exhibited a small semicircle at the high frequency regime with a R_CT_ value of 3.7 kOhm. After the self-assembly of 4-ABA on the surface of the SPEs, the Nyquist plot showed an increase in the width of the semicircle and the R_CT_ increased to 6.0 kOhm. Following immobilization of the polymeric receptors via an organic coupling reaction, a further increase to a R_CT_ = 7.9 kOhm was observed. This trend in increase in R_CT_ was in line with previous reports, which discussed that all the material deposited onto the SPEs is essentially non-conductive and thereby hampers electron transfer of the [Fe(CN)_6_]^3−/4−^ redox couple [36]. Thus, it confirmed the successful functionalization of nanoMIPs onto the SPEs using the described preparation method.

Figure 2B shows there was a minimal impact on the [Fe(CN)_6_]^3−/4−^ oxidation peak, which reduced from 7.4 × 10^−6^ A in a pure PBS solution to 6.1 × 10^−6^ A when there was 1 μM of LYZ in solution. This was a first indication that there was binding of LYZ to the nanoMIP layer since this was expected to block the electron transfer at the functionalized interface. However, CV is not the most sensitive electrochemical approach and we are interested in measuring trace amounts of LYZ in samples. Thus, experiments were performed with EIS since this technique has shown promise for highly selective and specific detection of small molecules using gold electrodes modified with nanoMIPs [37]. It was clear there was a significant increase in R_CT_ with increasing concentrations of LYZ, which was due to binding of the LYZ in the cavities of the nanoMIPs which leads to an increase in electrochemical resistance (Fig. 2C). A Randles circuit was used to construct the R_CT_ value at each concentration, with parameters of the circuit for each of the concentrations shown in Electronic Supplementary Material Figure S1. This demonstrated there was also a drop in capacitance when increasing the concentration of analyte, which can be attributed to a build-up of charges at the surface when LYZ binds.

**Fig. 3:**
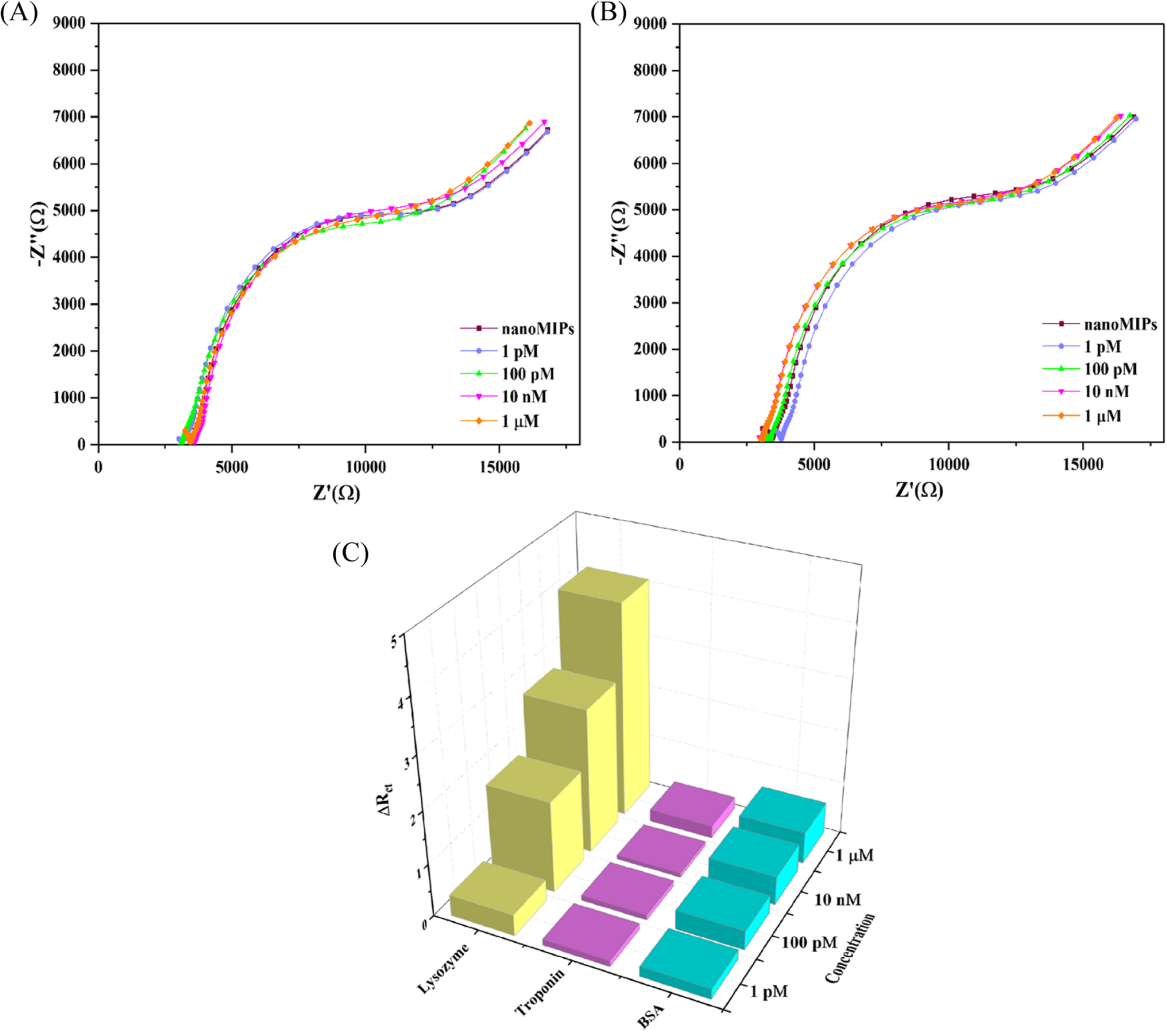
(**A**) EIS measurements with the Nyquist plots for the nanoMIP-immobilized SPE in the presence of various concentrations of BSA (1 pm to 1 µM) in PBS with 1 mM ferrocyanide, 1 mM ferrocyanide, and 0.1 M KCl and for troponin-I (**B**). In (**C**), the absolute change in R_CT_ vs the concentration of analyte is provided for LYZ (yellow), troponin-I (purple), and BSA (blue) as a bar chart

It was found that there was a significant increase in R_CT_ from 7.9 kOhm in a pure PBS solution to 12 kOhm in a LYZ concentration of 1 μM (19% increase). Using the three-sigma method and the slope of the calibration plot in the linear sensor regime (fit formula *y* = 0.6438*x* + 0.2885, with *R*^2^ = 0.994) to assess sensitivity, it was determined that the nanoMIP-based sensor can detect LYZ with a limit of detection of ~ 13 pM. This limit of detection is sufficient to measure trace LYZ concentration in food samples, thus confirming high specificity of the sensor. The selectivity of the sensor is further interrogated by exposing the nanoMIP-functionalized SPEs to proteins with similar functionality and isoelectric points. BSA was used as interference compound since it belongs to the same protein family as LYZ and is highly abundant in clinical samples such as blood [38]. Troponin-I was used since it is similar in molecular weight and, like LYZ, has a high isoelectric point [39]. Figure 3A demonstrates the response of the nanoMIP-functionalized SPEs to increasing concentrations of BSA (1 pm–1 μM) in PBS, while Fig. 3B showcases the results when solutions of PBS with increasing concentrations of troponin-I (1 pm–1 μM) were added. Contrary to when LYZ was added, there was no significant change in the Nyquist plot. This was further confirmed by determining the ΔR_CT_ values for these compounds, as shown in Fig. 3C. At a target concentration of 1 μM LYZ, a significant increase of 4 kOhm (19%) in R_CT_ was found, whereas for BSA this was only 0.6 kOhm and for troponin-I 0.2 kOhm. Thus, these results confirm the selectivity of our sensor system.

### Thermal detection of LYZ

For benchmarking the results obtained by electrochemical detections, measurements with an in-house build thermal device were performed. LYZ concentrations were thermally detected by mounting nanoMIP-functionalized SPEs into 3D-printed measurement cells to create an interface between the heat sink and the liquid reservoir. At first, the functionalized SPEs were left to stabilize for 15–20 min. Subsequently, PBS buffered solutions spiked with the target LYZ (0, 10 pM, 1 nM, 0.1 µM, 10 µM, and 1 mM), or proteins BSA and troponin-I in the same concentration range to evaluate selectivity, were manually added with a pipette. Following stabilization, the change in R_th_ was recorded and used to construct the corresponding dose–response curve since attachment of the target to the nanoMIP layer leads to a reduction of heat transfer at the solid–liquid interface (thus an increase in *R*_th_). Each SPE was used to measure the R_th_ at two different concentrations. After the analysis run, the addition cell was fully deconstructed and washed with DI water to ensure no residual sample solution could affect continuing runs.

**Fig. 4:**
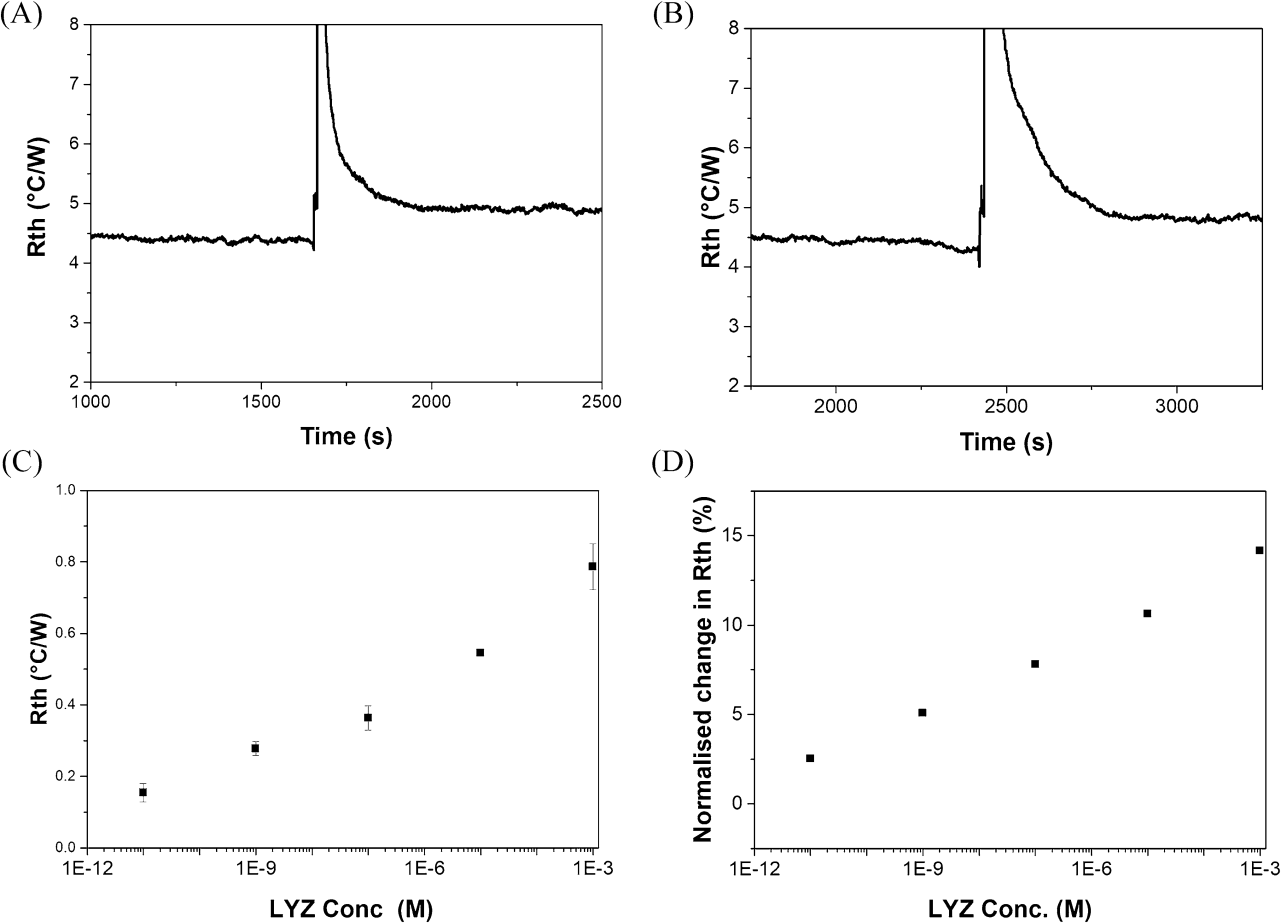
(**A**) Raw HTM data displaying transition from pure PBS to a solution of PBS spiked with 10 pM of LYZ. (**B**) Raw HTM data displaying transition from pure PBS to a solution of PBS spiked with 10 µM of LYZ. (**C**) Absolute change in R_th_ with increasing concentration compared to the R_th_ in pure PBS, for *n* = 2 measurements. (**D**) Normalized change in R_th_ with increasing concentration, which as calculated at R_th_ c = x divided over R_th_ at c = 0 and multiplied by 100 to get a percentage

Figure 4A displays the R_th_ over time from a stabilized PBS signal to an injection of LYZ solution (10 pm). It can be shown that the signal returned to the baseline value since only a small amount of LYZ was present and little binding to the polymer layer occurs. Figure 4B shows the R_th_ over time when a higher concentration of LYZ was present (10 µM). It can be observed that the PBS thermal signal stabilized at 1500 s, at a baseline of R_th_ = 4.38 ± 0.02 °C/W. The sharp increase witnessed at ~ 1900s was due to the addition of a PBS solution (at RT) spiked with LYZ. The PID feedback corrected for this change in temperature by increasing the voltage, thus leading in a spike of the R_th_. If no binding of the target would occur to the MIP layer on the surface of the SPE, the R_th_ would return to the same value before the addition of LYZ. However, binding of LYZ to the pores of the nanoMIP layer adds resistance to the surface, which leads to changes in thermal resistance as seen in Fig. 4B. This also confirmed that the higher the concentration of LYZ introduced to the nanoMIPs functionalized onto the surface of the electrode, the higher the response in terms of thermal resistance. At the highest concentration of 1 mM, an increase in nearly 0.7 °C/W was observed (~ 14% increase compared to the baseline value). A log scale was used to plot the absolute response in the mean R_th_ to increasing concentrations of LYZ in Fig. 4C from duplicate results. An individual standard deviation for each sample was calculated over one hundred data points (100 s of data). This standard deviation was derived from duplicate data sets and then used to act as the error for each data point (*n* = 2, mean standard deviation = 0.029).

In Fig. 4D, the dose–response curve is shown as the concentration vs the thermal resistance normalized to the starting value from the first individual data set. At 1 mM, the highest concentration of LYZ in PBS, an increase of ~ 14% in R_th_ was observed compared to a 2.5% increase on the injection of 10 pM. This was comparable to changes in R_th_ previously reported in literature [32, 36]. Using the linear range of the calibration curve (10 pM to 1 mM) and the standard deviation of the baseline (0.02 °C/W) as an average of all baseline SD values obtained throughout experimentation, a limit of detection of 1 fM was estimated according to the three-sigma method. Stabilization of a thermal resistance signal was achieved after a minimum of 15 min. A full sample run, composing of a blank and sample injection, was carried out in a duration of 30 min. While this thermal analysis yielded a lower LoD compared to EIS measurements, it required longer analysis time (30 min vs 5–10 min). In the future, it might be possible to calibrate the system thus only requiring an injection of a sample rather than stabilization in buffer.

### Proof-of-application via impedimetric determination of LYZ concentration in an egg white sample

**Fig. 5:**
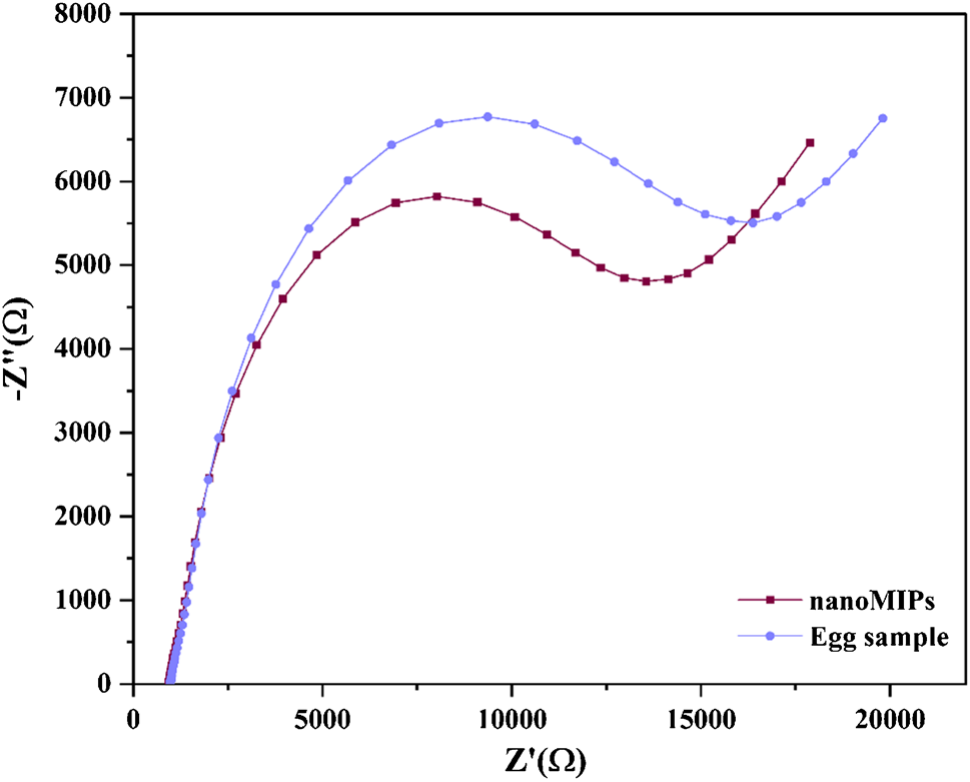
EIS measurements with the Nyquist plots for the nanoMIP-immobilized SPE in the presence of diluted egg sample in PBS with 1 mM ferrocyanide, 1 mM ferrocyanide, and 0.1 M KCl

An egg white sample was prepared as described in the “Proof-of-application impedimetric detection in an egg white sample” section, which was measured with a freshly prepared nanoMIP sensor to determine its LYZ content. Figure 5 shows the experimental impedance spectra for the nanoMIP-immobilized SPE in a buffered solution and after incubation in a diluted egg sample. By fitting data to the Randles circuit model, the normalized R_ct_ change was found to be 0.16%. Taking into account the dilution factor used in sample preparation, the total amount of LYZ found in the egg white was 2.7 mg mL^−1^. This concentration falls within the 2.5–4.5 mg mL^−1^ range of LYS concentration that is typical in chicken egg white [7]. Therefore, these analytical findings demonstrated that the sensor was successfully used to identify LYZ in intricate biological materials.

An overview of the specificity and measurement time of different point-of-care sensors used for LYZ detection is compared in Table 1.

**Table 1:** Comparison of different detection strategies for LYZ based either on aptamers or nanoMIPs. All the LoDs are based on measurements performed in spiked PBS solutions

Receptors	Detection technique	LoD	Analysis time	Reference
Aptamers	EIS	1 pM	5–10 min	[11]
Electroactive nanoMIPs	DPV	100 pM	5 min	[23]
Electropolymerized scopoletin-based MIPs	EIS	62 nM	5–10 min	[40]
nanoMIPs	EIS	13 pM	5–10 min	This manuscript
nanoMIPs	Thermal detection (HTM)	1 fM	30 min	This manuscript

Table 1 demonstrates that both aptamers and nanoMIP-based sensors coupled with electrochemical methods were capable of measuring LYZ at the relevant concentration range. However, LoDs should be compared in real food samples as these are complex matrices and fouling can have a significant impact on sensor specificity. Moreover, sample preparation, often involving dilution or extraction, is an important factor when deciding on the best sensing strategy. Stability should also be considered since nanoMIPs are generally more stable compared to aptamers due to their cross-linked nature, which is advantageous for measuring in complex matrices.

## Conclusion

We have developed a novel sensor for the electrochemical and thermal detection of LYZ, an allergic substance, using nanoMIP-based sensors. The nanoMIPs were synthesized using an innovative solid-phase approach, yielding uniform particles with a size of ~ 158 nm in the liquid state as determined by DLS in the liquid state and a size of 40–100 nM in the dry state as determined by SEM. Subsequently, these nanoMIPs were electrografted onto SPEs to construct disposable and low-cost sensors. The produced sensors exhibited a LoD of 13 pM for LYZ using EIS as read-out technique and 1 fM when employing the heat transfer method. Moreover, the nanoMIPs displayed excellent selectivity and no significant response was observed for proteins of similar molecular weight and isoelectric point (e.g. BSA and troponin-I). The obtained LoD values were sufficient to determine trace amounts of LYZ as food allergen and comparable to literature reports, while offering the advantages of fast measurement time (10-min EIS, 30-min thermal detection) and low-cost components. Considering the versatility of the polymeric receptors which can be adapted to virtually any analyte of interest, this creates significant commercial potential for nanoMIP-based test to improve food safety.

## Supplementary Information

Below is the link to the electronic supplementary material.

Supplementary file1 (DOCX 13 KB)
