# Supplementary material for: Electrochemical and thermal detection of allergenic substance lysozyme with molecularly imprinted nanoparticles
Source: Anal Bioanal Chem. 2023 Mar 11;415(18):4467–78. doi: 10.1007/s00216-023-04638-2 (PMC10329058; doi:10.1007/s00216-023-04638-2)
Supplement: Supplementary file 1 — Supplementary file1 (DOCX 13 KB) [file 216_2023_4638_MOESM1_ESM.docx]

**Supporting Information S-1:** The parameters for the fitted Randles circuit for the nano-MIP functionalized SPEs depending on the concentration of LYZ.

| **Concentration (pM)** | **R_s_ (ohm)** | **C_dl_ (F)** | **R_ct_ (ohm)** | **W (S*s^(1/2))** |
| --- | --- | --- | --- | --- |
| 0 | 1.88E+03 | 3.09E-05 | 7.91E+03 | 2.82E-04 |
| 1 | 1.90E+03 | 2.73E-05 | 8.31E+03 | 2.74E-04 |
| 10 | 1.57E+03 | 2.81E-05 | 8.45E+03 | 2.59E-04 |
| 100 | 1.77E+03 | 2.65E-05 | 9.64E+03 | 2.79E-04 |
| 1000 | 1.86E+03 | 2.60E-05 | 1.01E+04 | 2.76E-04 |
| 10000 | 2.03E+03 | 2.56E-05 | 1.06E+04 | 2.76E-04 |
| 100000 | 1.92E+03 | 2.25E-05 | 1.16E+04 | 2.69E-04 |
| 1000000 | 2.14E+03 | 2.21E-05 | 1.20E+04 | 2.67E-04 |
